# Recombinant EHV-1 Vector Expressing Immunodominant Hemagglutinin Protein of Equine Influenza Virus H3N8 (Sub-Lineage Florida Clade 2)

**DOI:** 10.3390/vaccines14070634

**Published:** 2026-07-20

**Authors:** Bidhan Chandra Bera, Manju Bernela, Aashwina Madhwal, Stephanie S. Pradhan, Venkataramireddy Balena, Taruna Anand, Supriya Kandasamy, Selvaraj Pavulraj, Wandit Ahlawat, Diksha Kandpal, Priya Mor, Gurmesh Bishnoi, Nishant Vasdev, Bhupendra Nath Tripathi, Tarun Kumar Bhattacharya, Nitin Virmani

**Affiliations:** 1 ICAR-National Research Centre on Equines, Sirsa Road, Hisar 125001, Haryana, Indiagurmesh0029@gmail.com (G.B.);; 2 Division of Pathology, ICAR-Indian Veterinary Research Institute, Bareilly 243122, Uttar Pradesh, India; 3 Department of Pathobiological Sciences, School of Veterinary Medicine, Louisiana State University, Baton Rouge, LA 70803, USA; 4 Indian Council of Agricultural Research, Krishi Bhawan, New Delhi 110012, Delhi, India

**Keywords:** EHV-1 vectored vaccine, bivalent vaccine, equine influenza virus, mutagenesis

## Abstract

Background: Equine herpesvirus type 1 (EHV-1) and equine influenza virus (EIV) are major respiratory pathogens in horses, causing significant economic losses in domesticated horses. Bacterial Artificial Chromosome (BAC) technology can be used to precisely manipulate the EHV-1 genome for the development of live-attenuated vector vaccines. Earlier, our group developed a live-attenuated EHV-1 vaccine by deleting virulence-associated genes using this technology and the mutant EHV-1 has been exploited for expressing foreign gene in the current study. Specifically, in this study, a mutant EHV-1 virus expressing the hemagglutinin (HA) gene of H3N8 EIV (sub-lineage: Florida clade 2) was generated and characterized in vitro. Methods: The HA gene of EIV (Florida clade 2) was used for antigen gene cloning. The expression cassette for the HA gene was commercially synthesized and inserted into the backbone of EHV1∆IR6 BAC using an *En passant* mutagenesis strategy. Recombinant clones were selected using antibiotic selection, PCR, and RFLP. Further, the recombinant virus was regenerated in RK-13 cells via transfection and characterized in vitro for plaque size, growth kinetics and immunofluorescence antibody test (IFAT). Results: PCR and RFLP confirmed the successful insertion of the HA gene into pEHV1∆IR6/gE BAC. The recombinant virus, vEHV1∆IR6/gE-HA(FC2), was successfully rescued in RK13 cells and demonstrated expression of the EIV haemagglutinin proteins by immunofluorescence assay. Although plaque size was reduced in the generated mutant virus in comparison to parental virus, the growth kinetics of the recombinant viruses were comparable to those of vEHV1∆IR6/gE. Conclusions: These findings demonstrate the successful expression of immunodominant hemagglutinin protein of EIV by recombinant EHV-1 and indicate the potential suitability of EHV-1 BAC as a vector platform for foreign gene expression.

## 1. Introduction

Equine herpesvirus (EHV) and equine influenza virus (EIV) are two important viral pathogens causing respiratory disease in horses. EHV-1 is ubiquitous in the horse population and belongs to the genus *Vercellovirus* in the *Alphaherpesvirinae* family, while EIV, one of the most important re-emerging respiratory pathogens, is caused by H3N8 influenza A virus [1]. EHV-1 and EIV pose significant health threats to domesticated horses, resulting in substantial economic losses to the equine industry [2,3]. In particular, EHV-1 causes a range of respiratory diseases, neurological disorders, and abortions in pregnant mares, while EIV leads to acute respiratory illness characterized by fever, cough, and nasal discharge [4,5]. Recently, the Texas Department of Agriculture issued an alert regarding EHV-1 outbreaks following the World Championship Barrel Racing Finals (Texas) because an aggressive strain of EHV-1 was reported by veterinary clinics in Central Texas [6]. Global surveillance data reveals inconsistent trends in equine influenza. High case numbers in the UK and the US during 2019–2020 declined thereafter, indicating successful, targeted control measures. Conversely, limited reporting from Asia and Africa suggests that the disease may be far more widespread than currently documented. Nevertheless, despite localized declines, the virus remains highly prevalent, as evidenced by persistent outbreaks and sporadic surges in cases across Europe and North America [7].

Furthermore, the ability of EHV-1 to establish latency and modulate host immune responses, including interferon, cytokine, and chemokine pathways, hinders the achievement of complete protection through vaccination [8]. The inherent challenges posed by frequent antigenic variation in equine influenza viruses and the complex immunopathology of equine herpesviruses underscore the need for novel vaccine approaches capable of providing broad and sustained protection against both pathogens simultaneously [9,10,11]. Currently available EHV-1 vaccines are mainly inactivated and modified live virus formulations. Inactivated vaccines reduce respiratory disease and abortion; however, they generally induce limited cell-mediated immune responses and require repeated booster immunizations [12]. At present, two licensed MLV vaccines are available in USA (Rhinomune^®^ by Boehringer Ingelheim) and Europe (Prevaccinol^®^ by MSD) [13,14]. However, available MLVs are unable to provide full protection to EHV1 infection. Despite wide use of such vaccines, disease outbreaks of EHV1 infections keep occurring globally, suggesting the need for the development of improved vaccines. Similarly, available equine influenza vaccines (EIVs) include inactivated whole-virus vaccines, subunit vaccines containing purified haemagglutinin antigen, and live-attenuated vaccines. Although these vaccines are widely used, they primarily stimulate antibody-mediated immune responses and often provide limited cross-protection against antigenically drifted strains [15]. This warrants the need of integrated approach of availability of combined vaccine for both the diseases which could mitigate the need for separate administrations of vaccines like ProteqFlu^®^ for EIV and Pneumabort-K^®^+1b for EHV, which are currently given individually [4]. 

Recently, herpesviruses have been exploited for the development of multivalent recombinant viral vectored vaccines capable of expressing heterologous antigens and inducing balanced humoral and cellular immune responses. Bacterial Artificial Chromosome technology has revolutionized EHV-1 vaccine design by enabling the precise and stable modification of the viral genome within efficient prokaryotic systems [16,17,18]. Specifically, this platform facilitates cloning of the complete genome of EHV-1, thereby enabling systematic deletion of virulence-associated genes and the integration of heterologous sequences to develop modified and vector vaccines [19,20]. BAC-based mutagenesis has also been successfully employed for precise engineering of live-attenuated EHV-1 vectors expressing immunodominant genes of various other viruses viz., H1N1 swine influenza virus [21], Bluetongue virus [20], Rift Valley Fever virus [22], and Canine Distemper Virus [23]. Earlier, our group had developed several mutant EHV-1 viruses by deleting virulence (IR6 and gE) and MHC-1 down regulation (pUL43 and pUL56)-associated genes in various combinations using BAC technology [24]. In the present study, the hemagglutinin (HA) gene of equine influenza virus (H3N8, sub-lineage-Florida clade 2) was inserted into the genome of mutant EHV1 to generate a recombinant virus that could be used as vaccine candidate against both the diseases. The equine influenza virus (H3N8) strain used in this study belongs to the Florida clade 2 sub-lineage, which is currently one of the predominant circulating lineages globally and is recommended for inclusion in contemporary vaccine formulations. Therefore, selection of the HA gene from this lineage ensures epidemiological relevance and enhances the translational potential of the recombinant vaccine construct.

## 2. Materials and Methods

### 2.1. Cells and Viruses

Previously generated single deletion mutant pEHV-1ΔIR6 BAC (vTOH) containing green fluorescent protein (gfp) was used to construct the recombinant virus carrying the HA gene of EIV (Florida Clade 2) in its backbone. The wild EHV-1 strain (vToH) was isolated from an abortion case in a mare from Tohana city in Haryana state of India, and EIV(H3N8) isolate [A/eq/jammu-Katra/06/08 (H3N8} obtained from horses in Katra city of Jammu and Kashmir state of India, were used for the studies. The EHV-1 isolate represents a clinically confirmed abortion case in 1996, while the EIV isolate originated from a respiratory disease outbreak in horses in the year 2008. The EIV isolate was previously characterized and confirmed to belong to the Florida clade 2 sub-lineage based on HA gene sequence analysis, supporting its epidemiological relevance for vaccine design. All viruses were propagated and maintained in the Pathology laboratory at ICAR-NRCE, Hisar. Rabbit Kidney (RK13) cells were maintained in DMEM supplemented with 10% FBS (Thermo Fisher Scientific, Waltham, MA, USA) and antibiotics (100 U/mL penicillin and 0.1 mg/mL streptomycin). The recombinant virus was propagated in RK-13 cells.

### 2.2. Design and Construction of Codon-Optimized HA Expression Cassette

The hemagglutinin (HA) gene of equine influenza A virus (H3N8 subtype) was codon-optimized for enhanced expression (CAI ≥ 0.85, GC 45–55%) in equine/mammalian cells using OptimumGene^TM^ algorithm (GenScript, Piscataway, NJ, USA). The expression cassette for the HA gene was commercially synthesized and cloned into the pUC57 vector. Subsequently, the clone was transformed into *E. coli* DH5α, selected on LB-ampicillin (100 µg/mL). Positive clones were verified by colony PCR. The isolated pUC57-HA plasmid was digested with AscI, followed by DpnI treatment. Finally, the released HA expression cassette was used for *en passant* mutagenesis strategy.

### 2.3. Generation of Recombinant Construct pEHV1∆IR6/gE-HA(FC2)

The previously generated IR6 deletion mutant of EHV-1 (pEHV1∆IR6) was used to insert a codon-optimized EIV-HA gene expression cassette using a two-step *en passant* Red recombination strategy, as described earlier [25]. The Red recombination method was carried out in *E. coli* strain-GS1783 harboring the mutant EHV1-BAC construct. The *E. coli* GS1783 is a modified strain of *E. coli* having encoded λ Red recombination genes (gam, bet, exo) and the I-SceI homing endonuclease expression system on the chromosome of the bacteria [26]. The λ Red genes are regulated by a temperature-sensitive promoter controlled by λ repressor. Therefore, the strain was routinely cultured at 30–32 °C to minimize Red expression and shifted to 41–42 °C for 15–20 min to induce the Red expression system for efficient recombineering. The encoded I-SceI enzyme is expressed under the control of arabinose-inducible promoter which is activated in the presence of arabinose in the culture media. The first Red recombination reactions were carried out by electroporating an HA expression cassette into GS1783 *E. coli* cells harboring the pEHV-1 ∆IR6 BAC to replace the ORF2 region of the EHV-1 genome. The positive colonies were screened by colony PCR. The confirmed first Red recombinant clones were subsequently subjected to the 2nd Red recombination strategy to remove the KAN cassette by inducing I-SceI expression with 1% arabinose. The KAN-sensitive clones were selected by replica plating and confirmed by colony PCR. 

Subsequently, the virulence gene gE was deleted from the recombinant EHV1 construct to generate a highly attenuated double-gene-deleted recombinant virus using *en passant* Red-mediated mutagenesis. The gE deletion cassette (KAN cassette) containing the kanamycin resistance marker and homologous flanking sequences of the gE gene was prepared by PCR amplification using the pLAY2 vector as a template and Phusion high-fidelity DNA polymerase. The KAN cassette was electroporated into *E. coli* harboring pEHV-1∆IR6-HA(FC2) BAC to replace the gE gene from the recombinant construct pEHV1∆IR6-HA(FC2) in Red1 mutagenesis, and subsequently, the KAN cassette was removed by Red2 recombination step. The KAN-sensitive clones were selected by replica plating and further confirmed by cPCR of the gE-deleted regions.

#### Restriction Fragment Length Polymorphism (RFLP) Analysis

Restriction fragment length polymorphism (RFLP) analysis was performed to confirm the generated recombinant construct-pEHV1∆IR6/gE-HA(FC2). The restriction enzyme—*Hind III* was selected for digestion of constructs based on in silico restriction mapping, which predicted distinct and readily resolvable fragment patterns. The digestion reactions were incubated at 37 °C for 1 h and resolved on a 0.8% agarose gel via electrophoresis at 45–50 V for 18–20 h. The gel was stained with ethidium bromide (0.5 µg/mL) for 1 h to facilitate nucleic acid visualization (Uvitec Essential V6 Gel Documentation System, Cambridge, UK).

### 2.4. Regeneration of Recombinant vEHV1∆IR6/gE-HA(FC2) Virus

For regeneration of recombinant virus, RK13 cells were seeded into 6-well plates (Greiner Bio-One, Kremsmünster, Austria) 24 h prior to transfection to achieve optimal confluency (60–70%). pEHV1∆IR6/gE-HA(FC2) plasmid DNA (1–2 µg) was combined with polyethyleneimine (PEI; 1 mg/mL in water) (Polysciences, Waarington, PA, USA) at a 1:1 ratio (µg DNA: µL PEI in 90 µL of OptiMEM (Gibco, Waltham, MA, USA). The mixture was incubated at room temperature for 30 min to allow complex formation. Following incubation, 500 µL of 2% MEM was added to the transfection mixture, which was then applied to the pre-washed RK13 cell monolayer and allowed to adsorb for 3 h. Subsequently, 1.5 mL of 2% MEM was added to each well to support continued cell growth. The plate was observed daily for the regeneration of the recombinant virus.

### 2.5. Evaluation of Stability and Growth Properties of the vEHV1∆IR6/gE-HA(FC2)

#### 2.5.1. Plaque Size Determination

To assess plaque morphology and plaque size, RK13 cell monolayers cultured in 6-well plates were infected with wild-type EHV-1, vEHV1∆IR6/gE, or vEHV1∆IR6/gE-HA(FC2) virus at a multiplicity of infection (MOI) of 1.0 and allowed for 1 h adsorption. Subsequently, the monolayers were washed gently, and overloaded with 1.5% methylcellulose medium. After 48 h of incubation at 37 °C, the cells were fixed and stained with 0.2% crystal violet solution. Approximately 50 plaques were imaged and measured using ImageJ (version 1.54g) software, as described previously [27].

#### 2.5.2. Growth Kinetics In Vitro

Wild-type EHV-1, vEHV1∆IR6/gE, and vEHV1∆IR6/gE-HA(FC2) were inoculated into RK-13 cell monolayers at an MOI of 0.01. After 1 h of adsorption, cells were washed and overlaid with 2% FBS supplemented Minimum Essential Medium (MEM) followed by incubation at 37 °C. Samples were harvested at 6, 12, 24, 36, 48, and 72 h post-infection and centrifuged at 3000 rpm for 10 min. Both supernatant and pellet were collected for extracellular and intracellular titer estimation, respectively. For quantification of viral titers, RK-13 cell monolayers were infected using ten-fold serial dilutions prepared from both intracellular and extracellular samples. Following viral adsorption, cultures were overlaid with a 1.5% methylcellulose solution to restrict viral spread across the monolayer. After 48 h of incubation, the cells were fixed with absolute ethanol and stained with 0.2% crystal violet to facilitate detection and enumeration of viral plaques, following established protocols [28].

#### 2.5.3. Indirect Immunofluorescence Assay

An indirect immunofluorescence assay was performed to assess HA gene expression. Briefly, porcine kidney (PK-15) cells were infected with vEHV1∆IR6/gE-HA(FC2) at an MOI of 0.01. After 1 h, the inoculum was removed, and cells were supplemented with MEM containing 10% FBS. Following 48 h of incubation, cells were fixed with 4% paraformaldehyde, permeabilized with 0.1% Triton X-100 for 10 min, and blocked with 1% bovine serum albumin (BSA). Hyperimmune rabbit serum raised against equine influenza virus was used as the primary antibody, whereas goat anti-donkey TRITC-conjugated antibody was used as the secondary antibody. Excess secondary antibody was removed by washing the cells three times, and fluorescent signals were visualized using an inverted fluorescence microscope.

### 2.6. Statistical Analysis

All experiments were performed in triplicate. Statistical differences were analyzed using one-way analysis of variance (ANOVA) followed by Tukey’s *t*-test for multiple comparisons (*p* < 0.05). Statistical analyses were performed using GraphPad prism version 8.0.2.

## 3. Results and Discussions

### 3.1. Construction of Recombinant EHV-1 Viruses Carrying Hemagglutinin Genes of EIV

**Figure 1:**
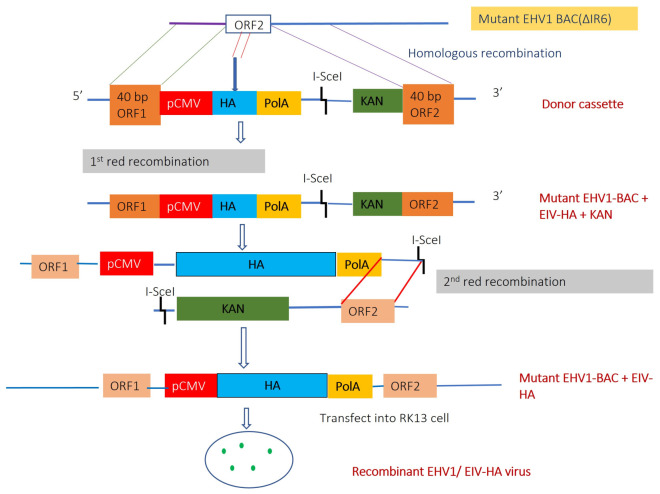
Strategy for insertion of HA expression cassette into mutant pEHV-1 ∆IR6 BAC.

The HA gene of EIV used in this study was fully characterized at sequence level earlier by our team, which confirmed its clustering within the Florida clade 2 lineage of H3N8 equine influenza viruses, consistent with previously reported circulating strains [29]. This supports the genetic relevance of the selected antigen for inclusion in the recombinant EHV-1 vector. The hemagglutinin gene from equine influenza virus H3N8 (sub-lineage: Florida clade 2) was selected for development of the expression cassette and was codon-optimized to achieve enhanced protein expression in equine cells. Studies have shown that codon optimization can significantly improve protein yields in heterologous expression systems [30,31], and specific modifications, such as increasing GC content, have been linked to improved immunogenicity and expression in DNA vaccines against avian influenza viruses [32]. Finally, the HA expression cassette was commercially synthesized and cloned into the pUC 57 vector with a CMV promoter, polyA tail sequence, I-SceI restriction site, kanamycin as a selection marker, and homology arms corresponding to ORF2 region of pEHV-1∆IR6 BAC. Figure 1 shows the strategy for insertion of this HA cassette into EHV-1 BAC.

**Figure 2:**
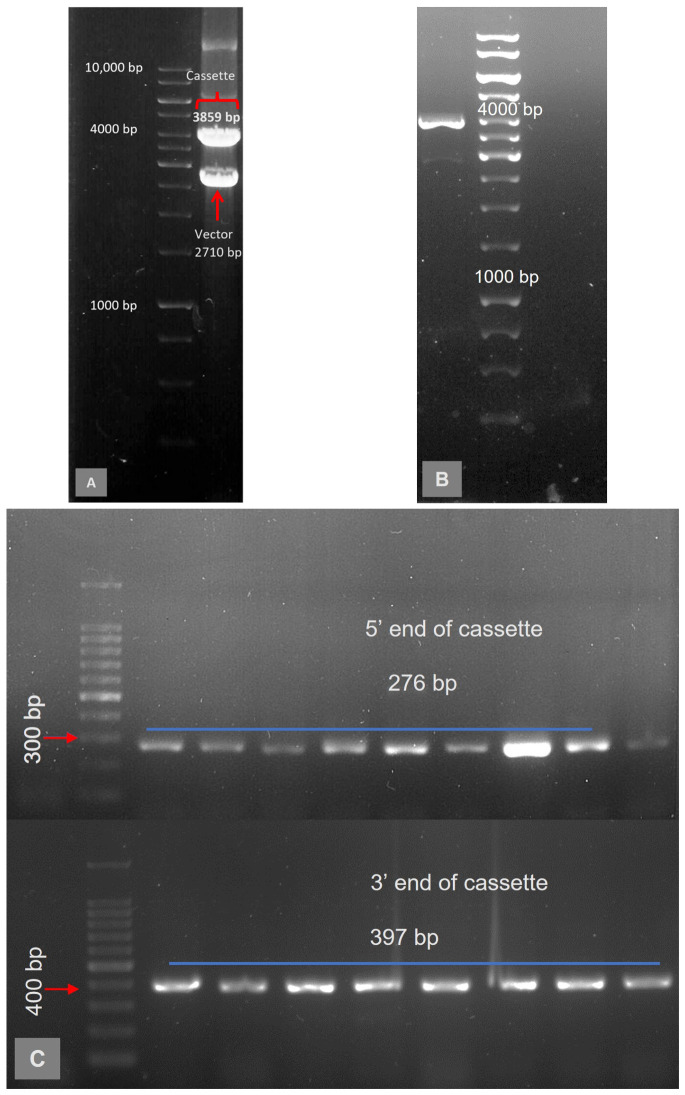

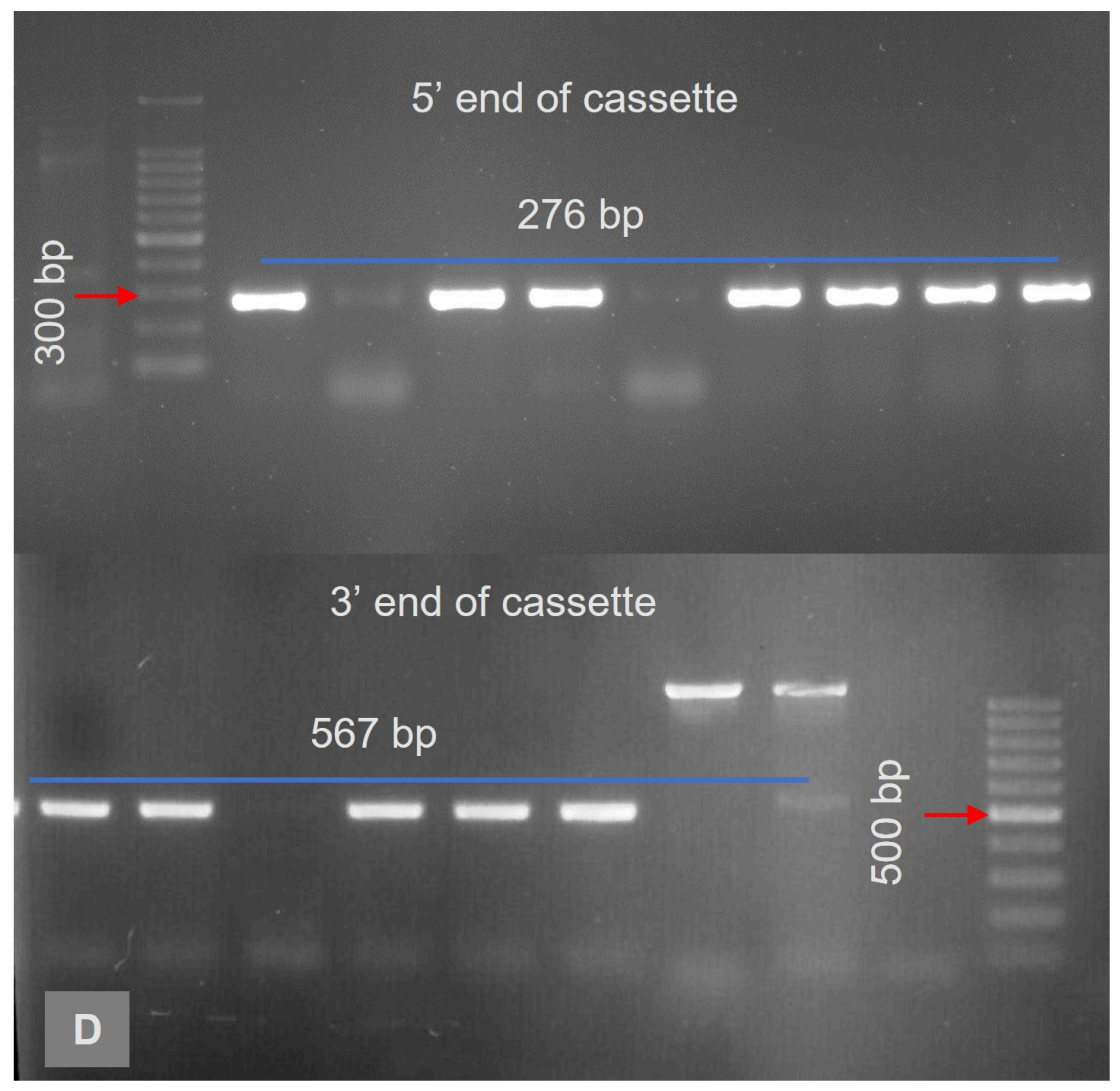
Strategy to generate recombinant clones of pEHV1∆IR6-HA (FC2). (**A**) RE digested pUC 57 vector showing release of cassette. (**B**) gel-purified HA expression cassette; (**C**) cPCR confirmation of Red1 clones of pEHV1∆IR6-HA (FC2); and (**D**) cPCR confirmation of Red 2 clones of pEHV1∆IR6-HA (FC2).

The HA expression cassette was released from the pUC 57 vector (Figure 2A), gel purified (Figure 2B), and electroporated into GS1783 electrocompetent cells harboring pEHV1∆IR6 BAC. The Red 1 positive clones generated amplicons of 276 and 397 bp at the 5’ and 3’ ends of the cassette, respectively (Figure 2C). Direct colony PCR using junction-specific primers for the target regions confirmed homologous recombination at the desired site while eliminating false positives. Subsequent Red 2 screening with cPCR primers yielded amplicons of 276 and 567 bp at the 5’ and 3’ ends of the cassette (Figure 2D), respectively, indicating precise I-SceI mediated excision.

**Figure 3:**
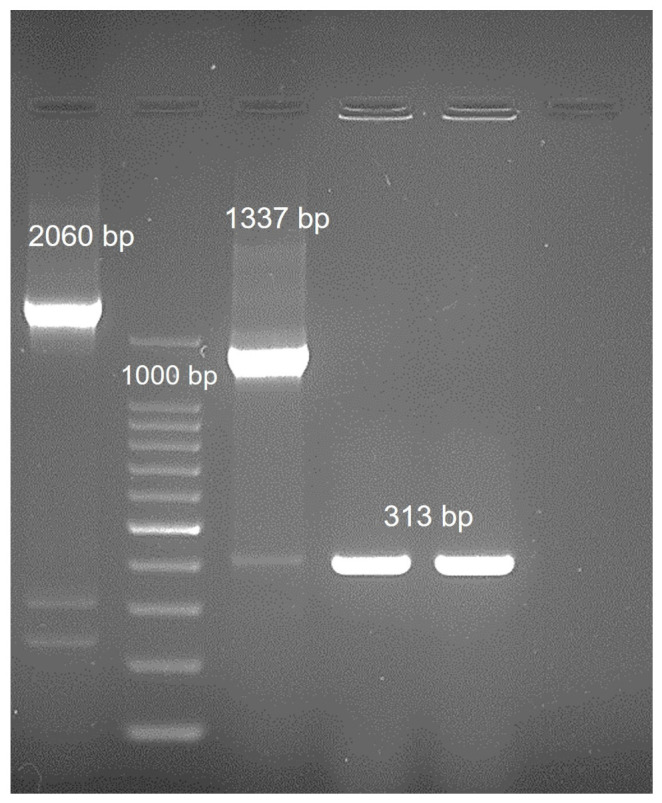
L1: Red 2 clone pEHV1∆IR6-HA (FC2) generating 2070 bp amplicon, L2: marker, L3: Red 1 clone of pEHV1∆IR6/gE-HA (FC2) generating 1337 bp amplicon, L4 and L5: Red 2 clone of pEHV1∆IR6/gE-HA (FC2) generating 313 bp amplicon.

Following this, the gE gene was deleted from the pEHV1∆IR6-HA (FC2) clones using the *en passant* mutagenesis strategy again. Figure 3 shows the agarose gel images of Red 1 and Red 2 clones of pEHV1∆IR6/gE-HA (FC2). The Red 1 clone generated a 2070 bp amplicon in gE intact clones, and a 1337 bp amplicon in gE-deleted clones due to KAN cassette insertion. In EHV1∆IR6/gE-HA (FC2) gE-deleted Red 2 clones, an amplicon of 313 bp was generated due to deletion of the KAN gene (~1 kB). The final clones obtained were designated pEHV1∆IR6/gE-HA (FC2). This dual-verification strategy mirrors earlier *en passant* protocols [25], where PCR prescreening reduced RFLP workload by 85% while maintaining mutagenesis fidelity.

**Figure 4:**
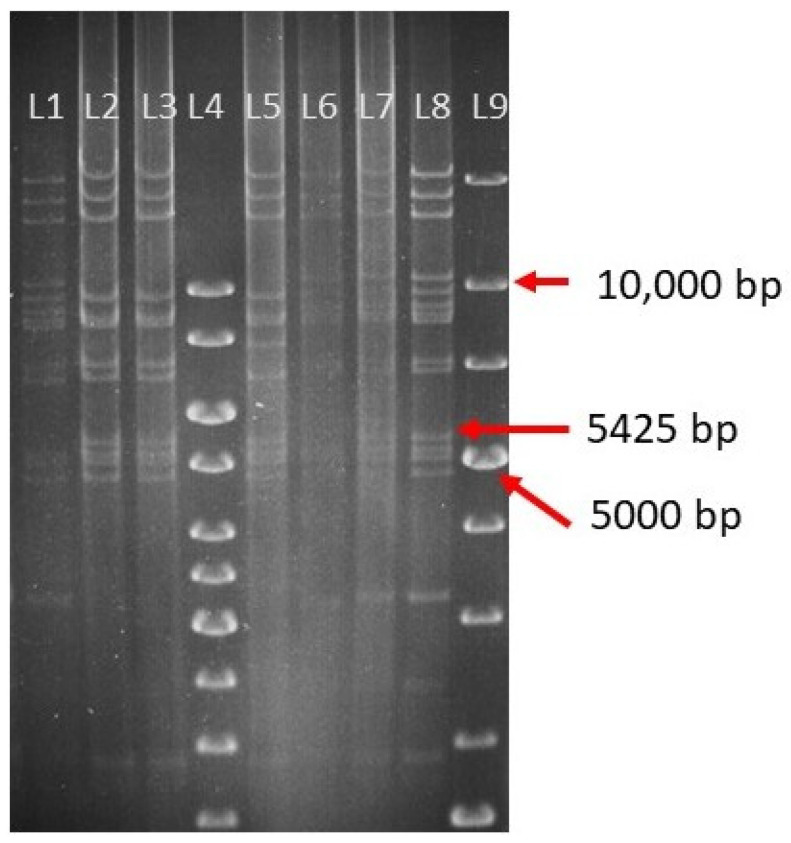
RFLP digestion profile of L1: pEHV1∆IR6/gE BAC, L2 and L3: pEHV1∆IR6/gE-HA(FC2), L4: marker, L5 to L8: pEHV1∆IR6/gE-HA(FC2), and L9: marker, using *HindIII* enzyme. Successful clones generated an additional fragment at 5425 bp in comparison to pEHV1∆IR6/gE.

Restriction fragment length polymorphism (RFLP) analysis provided definitive orthogonal confirmation of Red2 clone integrity, with Hind III digestion patterns precisely matching in silico predictions and distinguishing recombinant BACs from the parental double-deletion mutant. The *HindIII* restriction enzyme was used for analysis based on in silico prediction using NEBcutter, which generated distinct and easily resolvable fragment patterns. *HindIII* RFLP patterns of pEHV1∆IR6/gE BAC and pEHV1∆IR6/gE-HA(FC2) Red2 clones are shown in Figure 4. *HindIII* (RFLP) profiling of positive RED2 clones revealed an additional 5425 bp band corresponding to precise HA cassette integration, which was completely absent in the unmodified parental double-deletion mutant BAC. This diagnostic fragment addition, as predicted by NEBcutter simulation, further confirmed scarless recombination at the engineered locus, eliminating any residual selection markers and strictly excluding off-target recombination or parental contaminants, establishing genetic homogeneity prior to virus regeneration. Recombinant clones that matched the NEBcutter simulation results were selected for virus regeneration experiments. 

### 3.2. Regeneration of vEHV1∆IR6/gE-HA(FC2)

**Figure 5:**
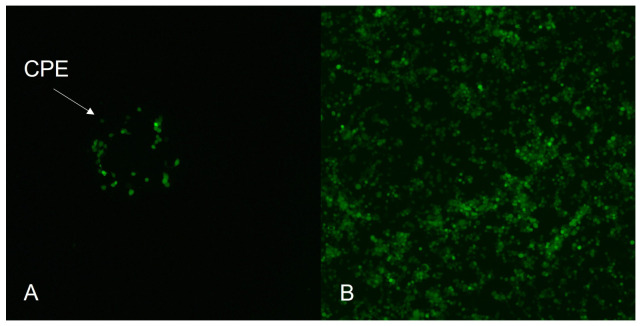
Virus regeneration pEHV1∆IR6/gE-HA(FC2). (**A**) Passage 1 and (**B**) passage 8.

For regeneration of recombinant virus from the pEHV1∆IR6/gE-HA(FC2), plasmid DNA was purified from successful recombinant clones and transfected into RK-13 cells. Cells expressing GFP were observed, and viral plaques became evident two days post-transfection. Figure 5 shows the regenerated vEHV1∆IR6/gE-HA(FC2) at different intervals. It was observed that the recombinant virus exhibited slow growth initially but was well-adapted over the passages, demonstrating complete cytopathic effect at 72 h. The adapted recombinant virus was then bulk cultured for further analysis of plaque morphology and intra/extracellular growth patterns.

This passage-dependent cytopathic effect is a well-documented phenomenon in BAC-derived herpesvirus recombinants. Similar observations were made earlier for delayed CPE in initial passages of *en passant*-generated pseudorabies virus, attributed to suboptimal virion maturation from miniF-excised BACs [25].

### 3.3. Stability and Growth Properties of the Recombinant Virus

#### 3.3.1. Plaque Size Estimation

**Figure 6:**
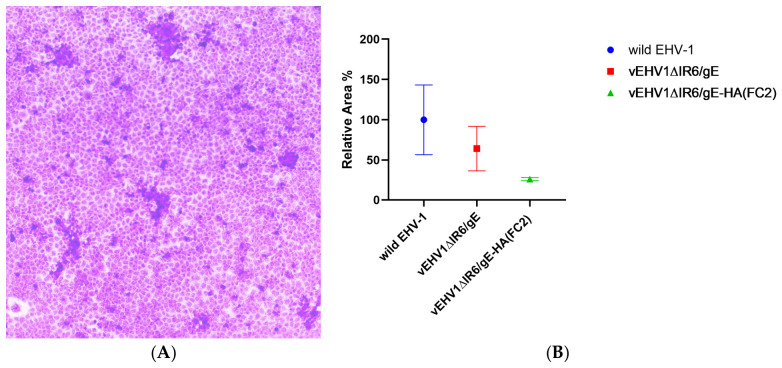
Plaque morphology of vEHV1∆IR6/gE-HA(FC2). (**A**) Representative figure of plaque formation. (**B**) Plot of reduction in the relative area of the plaques formed by different viruses (plaque diameter of wild EHV1 (vToH-BAC) was set to 100% and mean diameter ± SD were given).

The morphology of the plaque of the generated recombinant virus was evaluated to check the effect of insertion of the HA gene by replacing the ORF1 region of EHV1 in the spreading of the virus from cell to cell. The representative images of vEHV1∆IR6/gE-HA(FC2) plaques have been depicted in Figure 6A. The plaque diameters of the recombinant virus expressing the HA gene were measured and compared to those of the parental or wild-type virus. The plaque size of vEHV1∆IR6/gE-HA(FC2) was reduced by 74.12% in comparison to wild-type while vEHV1∆IR6/gE presented a 35.57% reduction (Figure 6B). Statistical analysis performed using one-way ANOVA revealed significant (*p* < 0.001) reduction in the plaque diameters of the recombinant viruses due to insertion of the HA gene. However, a 20% reduction in plaque size was observed in recombinant EHV1 harboring VP2 and VP5 genes of Bluetongue Virus [20].

This reduction reflects a defect in cell-to-cell dissemination rather than impaired replication as indicated by comparable extracellular and intracellular titers matching with parental replication efficiency of generated vEHV1∆IR6/gE-HA(FC2) virus.

#### 3.3.2. In Vitro Growth Kinetics

**Figure 7:**
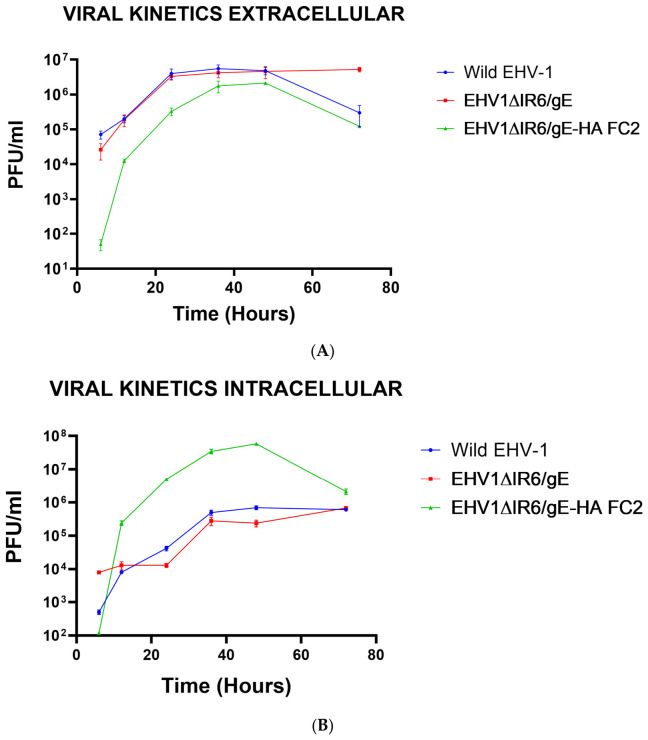
Growth kinetics of wild EHV-1,vEHV1∆IR6/gE, and vEHV1∆IR6/gE-HA(FC2) at different intervals. (**A**) Intracellular growth kinetics; (**B**) extracellular growth kinetics.

The replication efficiency and release characteristics of the vEHV1∆IR6/gE-HA(FC2) were quantitatively assessed by comparing its intracellular and extracellular viral titers in comparison to wild-type EHV-1 strain Tohana (vToH) in RK13 cells (Figure 7). At 36–48 h post-infection, the intracellular viral titers for the vEHV1∆IR6/gE-HA(FC2) were one log higher than wild-type (vToH) and vEHV1∆IR6/gE. Conversely, the extracellular titers of the insertion mutant were one log lower than those of the wild-type virus.

Despite these observed numerical trends, statistical analysis revealed no significant (*p* < 0.05) differences among any of the groups at all time intervals tested. This implies that, while the HA insertion might induce slight modulations in the balance between cell-associated and released virus, it does not critically compromise overall viral replication or egress efficiency in the RK13 cell line. Similar observations were also reported in studies of EHV1 based vector vaccine for canine influenza virus [33], H3N8 EIV (sub-lineage Florida clade 1) [34] and BTV [20].

The results of plaque morphology and growth kinetics revealed that vEHV1∆IR6/gE-HA(FC2) virus was slightly impaired in terms of cell-to-cell spread, but infectious virus production was not affected by the insertion and expression of the HA antigens. The multi-step growth kinetics primarily reflect overall viral replication efficiency over time, whereas plaque size is influenced by the efficiency of cell-to-cell spread. The reduced plaque size observed in the recombinant vEHV1∆IR6/gE-HA(FC2) virus may therefore indicate a modest alteration in virus spread dynamics rather than a defect in replication per se. This suggests that recombinant vEHV1∆IR6/gE-HA(FC2) virus can be easily manageable with regard to efficient handling for scale-up production.

### 3.4. Analysis of HA Protein Expression by Recombinant vEHV1∆IR6/gE-HA(FC2) Virus

**Figure 8:**
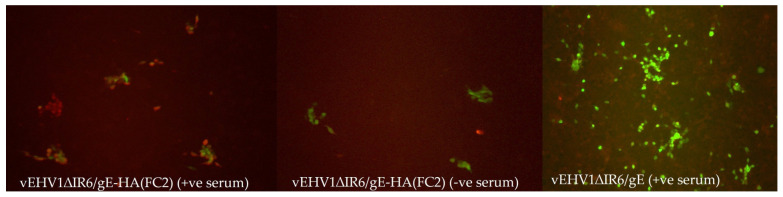
Immunofluorescence antibody test of vEHV1∆IR6/gE-HA(FC2).

The expression of HA protein by recombinant virus was evaluated by indirect immunofluorescence assay (IFA). The hyperimmune serum against equine influenza virus specifically detected HA protein of EIV (Florida clade 2) as a red fluorescence visual marker in cells infected with recombinant vEHV1∆IR6/gE-HA(FC2) virus, but not in the cells infected with wild virus (Figure 8). The recombinant viruses were continuously passaged up to passage 10 and continuously observed stable expression of HA protein throughout passages, as verified by IFA. The result indicates the capacity of the EHV1∆IR6/gE mutant to express heterologous genes, supporting its utility for vaccine development or gene delivery applications.

## 4. Conclusions

In the present study, a recombinant EHV-1 [(EHV-1ΔIR6/gE-HA(FC2)] virus expressing the hemagglutinin (HA) gene of H3N8 equine influenza virus (Florida clade 2) was successfully constructed and characterized in vitro. A reduction in plaque size was observed for the recombinant virus. Further, the IFAT results suggested successful expression of the HA gene. The recombinant virus exhibited stable HA expression, reduced plaque size, and growth kinetics comparable to the parental virus, indicating preserved replication competence despite genetic modification. These findings demonstrate the feasibility of the EHV-1 BAC platform for heterologous antigen expression; however, further studies are required to evaluate immunogenicity, safety, and protective efficacy in an in vivo model.

## Data Availability

The original contributions presented in this study are included in the article. Further inquiries can be directed to the corresponding authors.
